# Comparing Diabetes Management Knowledge Among Nursing Home Nurses in Spain and Poland: A Cross‐Sectional Study

**DOI:** 10.1155/nrp/5566896

**Published:** 2026-02-11

**Authors:** Ana Domínguez-Navarro, Juan Manuel Picardo-García, Consuelo López-Fernández, Natalia Pawlak, Lena Serafin

**Affiliations:** ^1^ Department of Nursing and Physiotherapy, Faculty of Nursing and Physiotherapy, University of Cádiz, Cádiz, Spain, uca.es; ^2^ Doctoral School, University of Cádiz, Cádiz, Spain, uca.es; ^3^ Department of Clinical Nursing, Faculty of Health Sciences, Medical University of Warsaw, Warsaw, Poland, wum.edu.pl; ^4^ Doctoral School, Medical University of Warsaw, Warsaw, Poland, wum.edu.pl

## Abstract

**Background:**

Older adults with diabetes living in nursing homes require complex care. Nurses play a central role in this care, yet limited evidence exists comparing their diabetes‐related knowledge across different countries. This study compares diabetes management knowledge among nursing home nurses in Cádiz, Spain, and Warsaw, Poland.

**Methods:**

A cross‐sectional, multicenter study was conducted with 198 nurses in Cádiz and 57 nurses in Warsaw. The adapted 14‐item Diabetes Management Knowledge Assessment Tool (DMKAT‐14) was used to measure knowledge. Sociodemographic and professional variables were also collected. Independent *t*‐tests, ANOVA, and multiple linear regression were used for analysis.

**Results:**

Mean DMKAT‐14 scores were higher in Cádiz (7.47 ± 1.74) than in Warsaw (6.05 ± 0.79), though both were suboptimal. No significant differences in knowledge were found based on gender, postgraduate education, or diabetes‐specific training. In Cádiz, no predictors were statistically significant. In Warsaw, age was the only significant predictor of higher knowledge scores (*B* = 0.057, *p* = 0.011).

**Conclusions:**

Diabetes management knowledge among nursing home nurses in Spain and Poland shows room for improvement. Strengthening diabetes‐specific training may help improve diabetes management in nursing homes across Europe.

**Summary**
• What does this paper contribute to the wider global clinical community?
◦ This paper highlights that diabetes management knowledge among nursing home nurses remains suboptimal in both Spain and Poland, despite formal education and training, signaling an international gap in preparedness for geriatric diabetes care.
◦ This paper shows that traditional training and postgraduate education do not strongly predict knowledge, highlighting the need to rethink continuing education frameworks.
◦ This paper identifies age as a significant predictor of knowledge in Poland, suggesting generational or experiential differences that may be relevant for workforce planning.

## 1. Introduction

Diabetes mellitus is one of the most prevalent chronic conditions among older adults and poses significant challenges for long‐term care systems globally [1]. In institutionalized settings such as nursing homes, diabetes management is particularly complex due to frailty, polypharmacy, cognitive impairment, and functional limitations common in this population [2, 3]. These factors increase vulnerability to hypoglycemia, treatment‐related complications, and poor quality of life—requiring a highly individualized and coordinated care approach [1, 4].

In Europe, an estimated 21.8% of nursing home residents live with DM, with rates reaching 26.4% in the province of Cádiz, Spain [5, 6]. Compared to their community‐dwelling counterparts, institutionalized older adults with DM experience higher rates of cognitive decline, nutritional challenges, and reduced capacity for self‐management [3, 7]. These complexities underscore the essential role of nurses in diabetes care in nursing homes.

Nurses are responsible for daily glycemic monitoring, medication and insulin administration, recognizing hypo/hyperglycemic episodes, and adapting care plans to residents’ individual needs [8, 9]. They also play a vital role in educating residents and families, fostering adherence to therapeutic plans, and promoting autonomy and quality of life. However, their capacity to provide optimal diabetes care is often limited by high workloads, institutional constraints, and a lack of targeted training in geriatric diabetes management [10, 11].

Although international guidelines, including those from the American Diabetes Association (ADA), provide dedicated recommendations for managing diabetes in older adults within long‐term care and skilled nursing facilities, the implementation of these guidelines can be hindered by institutional constraints [1]. Factors such as rigid organizational routines, staffing shortages, and cultural norms can hinder the implementation of individualized care practices in nursing homes [12, 13].

Despite the critical role of nurses, few studies have examined their knowledge of diabetes care within nursing homes, particularly in comparative international contexts. Existing research is primarily focused on individual countries, leaving a gap in cross‐cultural understanding of how nursing education, experience, and health system structures influence diabetes management knowledge [14, 15].

To address this gap, the present study compares diabetes management knowledge among nursing home nurses in Cádiz, Spain, and Warsaw, Poland—two European regions with distinct sociodemographic, educational, and institutional characteristics. By examining differences in knowledge levels and associated factors, this research aims to identify strengths and areas for improvement in nursing practice and inform targeted educational interventions and policy development.

## 2. Methods

### 2.1. Study Design and Setting

This was a multicenter, observational, cross‐sectional study conducted among registered nurses working in nursing homes in Cádiz, Spain, and Warsaw, Poland. The study was reported according to the STROBE guidelines (see Supporting Table S1) [16].

### 2.2. Participants

Eligible participants were registered nurses currently employed in nursing homes, with direct care responsibilities for residents with diabetes mellitus and at least 6 months of professional experience in this setting. The 6‐month threshold was established to ensure that participants had adequate exposure to the institutional routines, patient profiles, and clinical responsibilities associated with diabetes care in nursing homes. Nurses without direct involvement in diabetes care, such as nurse managers or nurses in exclusively administrative roles, were excluded. Newly graduated nurses with less than 6 months of clinical experience in nursing homes were also excluded, as their limited exposure might not yet reflect routine clinical practice in this setting.

Data collection was conducted in Warsaw between March and June 2023 and in Cádiz between September and November 2023. A purposive sampling strategy was employed. A total of 204 nurses were recruited in Cádiz (198 eligible for analysis) and 57 in Warsaw.

The difference in sample size between Cádiz and Warsaw reflects the number and size of nursing homes accessible during the data collection period in each region, as well as differences in institutional participation and workforce availability.

### 2.3. Measurement Instrument

The primary instrument used to assess nurses’ knowledge was the Diabetes Management Knowledge Assessment Tool (DMKAT), originally developed by Modic et al. [17]. The DMKAT evaluates knowledge related to inpatient diabetes management, drawing upon clinical guidelines from the ADA and the American College of Endocrinology.

The DMKAT‐14 specifically assesses diabetes management knowledge, that is, knowledge related to the clinical management of diabetes in nursing home residents, rather than general or theoretical knowledge about diabetes.

To tailor the instrument to long‐term care settings, the original questionnaire was adapted by the principal investigator in collaboration with Dr. Mary Beth Modic. This process led to the creation of a 17‐item version (DMKAT‐17) focused on nursing home contexts. The instrument was subsequently translated and culturally adapted into Spanish and Polish using the ten‐step methodology proposed by Wild et al. [18]. During content validation, 13 Spanish experts reviewed the adapted tool. Three items (1, 2, and 6) were removed for not meeting the minimum threshold (content validity ratio [CVR’] > 0.58), resulting in a 14‐item version (DMKAT‐14). The Spanish version demonstrated strong content validity (content validity index [CVI] = 0.841), and the Polish version underwent equivalent adaptation procedures to ensure conceptual and semantic alignment.

Content validity was assessed using the modified Lawshe method proposed by Tristán‐López [19]. According to this approach, items with a modified CVR’ equal to or greater than 0.58 are considered acceptable. Only items meeting this criterion were retained, and the overall CVI was subsequently calculated as the mean of the acceptable items, following the original procedure proposed by Lawshe [20].

The DMKAT‐14 consists of 14 multiple‐choice items, each with one correct answer, and assesses nurses’ knowledge across four key domains relevant to diabetes care in nursing home residents: hyperglycemia, insulin therapy, prevention and management of hypoglycemia, and survival skills education. Each correct response is scored as one point, resulting in a total score ranging from 0 to 14, with higher scores indicating greater diabetes management knowledge. The instrument is not a Likert‐type scale but an objective knowledge assessment. According to Modic et al. [17], a mean score of 11 or higher is considered acceptable, reflecting approximately 80% of the knowledge required for effective diabetes management in nursing home settings. Completion of the questionnaire requires approximately 15 min.

In addition to the DMKAT‐14 responses, data were collected on sociodemographic characteristics (e.g., age, gender), educational background (e.g., degree level, postgraduate training), professional experience (e.g., total years, nursing home experience, diabetes‐specific experience), and personal or familial exposure to diabetes. Postgraduate training was defined as any formal education completed after obtaining the undergraduate nursing degree, including nursing specialties, university master’s degrees, and expert diplomas. Personal exposure to diabetes was defined as having diabetes oneself or having a close family member or friend diagnosed with diabetes.

### 2.4. Data Analysis

Statistical analyses were performed using IBM SPSS Statistics version 24. Descriptive statistics (means, standard deviations, frequencies, and percentages) were calculated for all variables. Independent samples *t*‐tests were used to examine differences in DMKAT‐14 scores by sex, level of university education, specific training, and personal or social exposure to diabetes. One‐way analysis of variance (ANOVA) was conducted to assess differences based on postgraduate education and personal exposure to diabetes. Effect sizes were calculated using Cohen’s d for *t*‐tests and eta squared (*η* ^2^) for ANOVAs.

University education (Diploma vs. Bachelor’s degree) was included as an independent variable only in the Cádiz model, reflecting the coexistence of both educational pathways in the Spanish nursing workforce following the transition from diploma‐ to degree‐based education. In the Warsaw sample, all participants held a bachelor’s degree; therefore, this variable showed no variability and was not included in the regression analysis.

Finally, separate multiple linear regression models were constructed for the Cádiz and Warsaw samples to identify predictors of diabetes knowledge scores. Independent variables included age, sex, educational level, years of experience, diabetes‐specific training, and personal exposure to diabetes. Statistical significance was defined as *p* < 0.05.

### 2.5. Ethical Considerations

The study received ethical approval from the Ethics Committee for Research in Cádiz, Spain (PEIBA0652‐N‐23), and the Ethics Committee of the Medical University of Warsaw (AKBE/66/2023). Informed consent was obtained from all participants. All procedures were conducted in accordance with the ethical standards of the Declaration of Helsinki.

## 3. Results

The study included 198 nurses in Cádiz and 57 in Warsaw. Most participants were women (84.3% in Cádiz and 86% in Warsaw), reflecting the gender distribution of the nursing workforce in both countries. The average age of nurses in Cádiz was 38.86 years (SD = 10.44), with a mean of 12.77 years (SD = 9.54) of professional experience. Nurses from Warsaw were older, with a mean age of 46.88 years (SD = 8.77), and had 21.27 years of experience (SD = 10.13). In both groups, the majority had no postgraduate education (56.6% in Cádiz; 63.2% in Warsaw). Specific training in diabetes was more frequent in Cádiz (34.3%) than in Warsaw (22.8%).

The mean DMKAT‐14 knowledge score was 7.47 (SD = 1.74) in Cádiz and 6.05 (SD = 0.79) in Warsaw, corresponding to approximately 53% and 43% of the maximum possible score, respectively, indicating higher diabetes‐related knowledge among nurses in Cádiz. Nevertheless, knowledge levels in both groups remained suboptimal (Table 1).

**TABLE 1:** Sociodemographic and professional characteristics of nurses in Cádiz and Warsaw.

Variable		Cádiz (*n* = 198)	Warsaw (*n* = 57)
Age (mean ± SD)		38.86 ± 10.44	46.88 ± 8.77

Professional experience (years)		12.77 ± 9.54	21.27 ± 10.13

Experience in nursing homes (years)		9.07 ± 8.24	18.52 ± 9.83

Experience in diabetes care (years)		10.56 ± 8.93	16.73 ± 9.76

Gender (%)	Women	167 (84.3)	49 (86.0)
	Men	31 (15.7)	8 (14.0)

Postgraduate training (%)	Yes	112 (43.4)	21 (36.8)
	No	86 (56.6)	36 (63.2)

Diabetes training (%)	Specific diabetes training	68 (34.3)	13 (22.8)
	No diabetes training	130 (65.7)	44 (77.2)

Personal or social exposure to diabetes (%)	Personal diabetes	7 (3.5)	0.0
	Family member with diabetes	76 (38.4)	15 (26.3)
	Friend with diabetes	22 (11.1)	38 (66.7)
	Family & friend with diabetes	12 (6.1)	0.0
	No known people with diabetes	81 (40.9)	4 (7.0)

Mean DMKAT‐14 Score		7.47 ± 1.74	6.05 ± 0.79

*Note:* Data are presented as mean ± standard deviation or *n* (%). Percentages were calculated using the total sample size for each city (Cádiz: *n* = 198; Warsaw: *n* = 57). Some variables may not sum to 100% due to rounding. No missing data were observed for the variables included in this table. DMKAT‐14 scores range from 0 to 14, with higher scores indicating greater diabetes management knowledge.

### 3.1. *t*‐Tests and ANOVA

In both cities, gender was not significantly associated with DMKAT‐14 scores. In Cádiz, mean scores were 7.61 for men and 7.45 for women, with a negligible effect size (Cohen’s *d* = −0.09; *p* = 0.315). In Warsaw, mean DMKAT‐14 scores were 6.10 for women and 5.75 for men; however, this difference was not statistically significant (*p* = −0.245).

Likewise, no significant differences were observed in DMKAT scores according to university degree level (Diploma vs. Bachelor’s degree) or diabetes‐specific training in either city (Table 2).

**TABLE 2:** Bivariate analyses of participant characteristics associated with DMKAT‐14 scores in Cádiz and Warsaw.

Variable	Group	City	*n*	Mean (SD)	Statistical test	*p* value	Effect size
Gender	Women	Cádiz	167	7.45 (1.74)	*t*‐test	*p* = 0.315	*d* = −0.09
	Men	Cádiz	31	7.61 (1.65)			
	Women	Warsaw	49	6.10 (0.80)	*t*‐test	*p* = 0.245	*d* = 0.79
	Men	Warsaw	8	5.75 (0.71)			

Postgraduate Education	No postgraduate	Cádiz	86	7.45 (1.73)	ANOVA	*p* = 0.433	*η* ^2^ = 0.030
	Nursing specialty	Cádiz	21	7.33 (1.49)			
	University master’s	Cádiz	9	8.44 (1.94)			
	University expert	Cádiz	61	7.31 (1.93)			
	> 1 Master’s/Expert	Cádiz	21	7.71 (1.16)			
	No postgraduate	Warsaw	36	6.17 (0.74)	ANOVA	*p* = 0.220	*η* ^2^ = 0.055
	University master’s	Warsaw	17	5.94 (0.83)			
	> 1 Master’s	Warsaw	4	5.50 (1.00)			

Diabetes Training	No training	Cádiz	130	7.34 (1.83)	ANOVA	*p* = 0.364	*η* ^2^ = 0.022
	Nonaccredited course	Cádiz	12	7.92 (1.62)			
	Accredited course	Cádiz	56	7.70 (1.50)			
	No training	Warsaw	44	6.05 (0.75)	ANOVA	*p* = 0.901	*η* ^2^ = 0.000
	Yes	Warsaw	13	6.08 (0.95)			

Personal or social exposure to diabetes	No known persons	Cádiz	81	7.23 (1.81)	ANOVA	*p* = 0.126	*η* ^2^ = 0.036
	Friend with diabetes	Cádiz	22	7.14 (1.83)			
	Family member	Cádiz	76	7.75 (1.71)			
	Both family and friend	Cádiz	12	7.33 (1.16)			
	Self (DM/gestational)	Cádiz	7	8.57 (1.13)			
	No known persons	Warsaw	4	6.25 (1.71)	ANOVA	*p* = 0.735	*η* ^2^ = 0.011
	Friend with diabetes	Warsaw	38	6.08 (0.54)			
	Family member	Warsaw	15	5.93 (1.03)			

*Note:* Independent‐samples *t* tests were used for binary variables, and one‐way analyses of variance (ANOVA) for variables with more than two categories. Results are reported using group means, standard deviations, *p* values, and effect sizes. Effect sizes are reported as Cohen’s d for *t* tests and eta squared (*η* ^2^) for ANOVA.

ANOVA was conducted to examine whether DMKAT‐14 scores varied according to postgraduate education, diabetes‐specific training, and personal exposure to diabetes.

In Cádiz, no significant differences in knowledge scores were observed based on postgraduate education level (*F* (6, 191) = 0.990, *p* = 0.433, *η* ^2^ = 0.030). Mean scores ranged from 7.23 among those without any known personal diabetes exposure to 8.57 among those who had diabetes themselves. Similarly, there were no significant differences in knowledge scores based on personal exposure to diabetes (*F* (4, 193) = 1.824, *p* = 0.126, *η* ^2^ = 0.036) or diabetes‐specific training (*F* (4, 193) = 1.089, *p* = 0.364), though nurses who had completed accredited or nonaccredited diabetes courses tended to score slightly higher (Table 2).

In Warsaw, the pattern was similar. Knowledge scores did not differ significantly by postgraduate education (*F* (2, 54) = 1.558, *p* = 0.220, *η* ^2^ = 0.055), diabetes‐specific training (*F* (1, 55) = 0.016, *p* = 0.901, *η* ^2^ = 0.000), or personal exposure to diabetes (*F* (2, 54) = 0.310, *p* = 0.735, *η* ^2^ = 0.011). Across all comparisons, effect sizes were small, and confidence intervals for group means overlapped, suggesting minimal practical differences (Table 2).

### 3.2. Linear Regression Analysis

In Cádiz, none of the independent variables significantly predicted DMKAT‐14 scores in the multiple linear regression models. The strongest trend was found for university degree level (*B* = 0.708, *p* = 0.054) and personal exposure to diabetes (*B* = 0.203, *p* = 0.072), both marginally nonsignificant. Age, sex, postgraduate education, specific diabetes training, and professional experience (general, in nursing homes, or in diabetes care) were not significant predictors (Table 3).

**TABLE 3:** Multiple linear regression models predicting DMKAT‐14 scores.

Variable	Cádiz (*B*, *p*)	Warsaw (*B*, *p*)
Age	−0.001, *p* = 0.952	0.057, *p* = 0.011^∗^
Sex	−0.002, *p* = 0.995	−0.384, *p* = 0.214
University education	0.708, *p* = 0.054	—
Postgraduate training	−0.045, *p* = 0.502	−0.134, *p* = 0.089
Diabetes‐specific training	0.151, *p* = 0.279	−0.001, *p* = 0.997
Years of professional experience	0.029, *p* = 0.370	−0.042, *p* = 0.112
Years in nursing homes	−0.014, *p* = 0.664	0.005, *p* = 0.836
Years with diabetic patients	0.007, *p* = 0.842	0.009, *p* = 0.617
Personal or social exposure to diabetes	0.203, *p* = 0.072	−0.330, *p* = 0.098

*Note:* Values represent unstandardized regression coefficients (*B*) with corresponding *p* values. Regression models were estimated separately for Cádiz and Warsaw. Model fit statistics (*R* ^2^, adjusted *R* ^2^, and *F* statistics), as well as standard errors and 95% confidence intervals, were examined during the analysis but are not shown to preserve table clarity. These results are available upon request. *p* < 0.05.

^∗^ *p* < 0.05.

In Warsaw, age was the only significant predictor of diabetes knowledge (*B* = 0.057, *p* = 0.011), indicating that older nurses had higher scores. Other variables such as postgraduate education (*B* = −0.134, *p* = 0.089) and personal exposure to diabetes (*B* = −0.330, *p* = 0.098) showed marginal associations but were not statistically significant. Years of experience, diabetes‐specific training, and sex were not associated with higher knowledge scores (Table 3).

## 4. Discussion

This study found that diabetes management knowledge among nursing home nurses was insufficient in both Cádiz and Warsaw, with mean DMKAT‐14 scores of 7.47 (SD = 1.74) and 6.05 (SD = 0.79), respectively. These low scores are particularly relevant in the context of long‐term care, where nurses are primarily responsible for managing complex diabetes needs in frail, older residents [9].

These findings are consistent with previous research identifying widespread knowledge gaps in diabetes care across long‐term care settings. Studies from Canada [10], New Zealand [11], and Norway [15] have reported similar deficits in blood glucose monitoring, insulin management, and the ability to tailor care to individual needs.

Our findings help contextualize previous evidence showing that nurses and other healthcare providers recognize gaps in their diabetes‐related competencies, particularly in areas such as insulin titration, hypoglycemia prevention, and diabetes management in the context of dementia or multiple comorbidities [21, 22]. Importantly, these studies also report a mismatch between healthcare providers’ perceived confidence and their objectively measured knowledge, suggesting that awareness of educational needs does not necessarily translate into adequate competence [23, 24]. This observation is consistent with our results, which showed no strong association between prior diabetes training or postgraduate education and higher knowledge scores. Together, these findings suggest that existing educational approaches may be insufficiently aligned with the specific clinical demands of diabetes care in long‐term care settings.

When educational interventions are delivered, they appear effective. Lega et al. [25] demonstrated that tailored programs can significantly improve knowledge and self‐confidence among nurses, dietitians, and personal support workers in long‐term care settings. However, sustaining this improvement remains difficult in environments characterized by high workloads, limited staffing, and frequent turnover [26].

Systemic problems also persist at the organizational level. As Sinclair et al. [27] observed, many nursing homes lack standardized diabetes protocols, audit mechanisms, or even designated diabetes leads. Inconsistent blood glucose monitoring, infrequent HbA1c testing, and minimal involvement of residents in self‐care are all common. These gaps may contribute to preventable complications such as hypoglycemia—a frequent and dangerous event in frail older adults with polypharmacy, impaired renal function, and diminished symptom awareness [28].

Hypoglycemia has been shown to affect health‐related quality of life (QoL) in older adults with diabetes as much as—or even more than—other diabetes‐related complications [29]. This is particularly concerning in institutionalized settings, where older adults often experience a progressive decline in QoL due to the chronic burden of diabetes [30] and the negative impact of institutional living itself [31]. Despite its clinical relevance, hypoglycemia remains a knowledge gap among healthcare providers. A hospital‐based study found that only about 40% of professionals correctly identified hypoglycemia as blood glucose levels below 3.9 mmol/L [21]. Moreover, providers in the same setting reported a strong need for further education on the recognition and management of blood glucose extremes, including the development of treatment plans for both hypo‐ and hyperglycemia [22].

Continuous glucose monitoring (CGM) may be a valuable strategy for identifying hypoglycemia among insulin‐treated residents in long‐term care settings [1]. Three studies have examined CGM use in nursing homes and subacute rehabilitation facilities, focusing on its feasibility and clinical utility [32–34]. Collectively, these findings suggest that CGM can successfully detect frequent and asymptomatic hypoglycemic episodes in institutionalized older adults and is generally well accepted by nursing staff. However, the implementation of CGM once again highlights the issue of insufficient training. While CGM has the potential to reduce the burden of fingerstick monitoring and facilitate more informed clinical decisions [35], its effectiveness depends heavily on adequate staff education [1]. In fact, healthcare providers have consistently reported a need for more knowledge related to diabetes technologies, including CGM, to confidently integrate such tools into daily care practices [22].

Interestingly, no strong associations were found between diabetes knowledge and variables such as education level, years of experience, or prior training in either Cádiz or Warsaw. This suggests that traditional forms of education and training may be insufficient or poorly aligned with the specific demands of diabetes care in nursing homes. For instance, in Poland, undergraduate nursing programs span a minimum of six semesters and require at least 180 ECTS credits and 4,720 h of theoretical and practical training as part of undergraduate nursing education. These programs also include English language training at a B1 level, potentially facilitating access to international guidelines and scientific literature [36]. In Spain, the nursing curriculum extends over 4 years with 240 ECTS credits and includes a mandatory foreign language component [37]. However, despite these substantial academic structures, the persistently low knowledge scores observed in both contexts highlight a gap between formal education and the practical competencies needed for delivering high‐quality diabetes care in long‐term care environments.

Although Polish nurses were older and had greater professional experience than their Spanish counterparts, this did not translate into uniformly higher diabetes knowledge. While age emerged as a significant predictor of knowledge in the Warsaw sample, years of professional experience did not, suggesting that in nursing home contexts, experiential exposure alone may be insufficient to ensure up‐to‐date, evidence‐based diabetes knowledge. This finding may be partly explained by the specific characteristics of the long‐term care environment. Evidence from other healthcare settings indicates that both work context and accumulated experience can influence diabetes knowledge. For example, hospital‐based nurses have been shown to demonstrate higher levels of diabetes knowledge than those working in other care settings, highlighting the role of clinical environment in shaping competencies [38]. Similarly, studies conducted in public health facilities report that longer professional experience is associated with higher levels of actual diabetes knowledge [23]. Taken together, these findings support the interpretation that the comparatively lower impact of professional experience observed in our study may reflect the nursing home context, where clinical routines may evolve more slowly and opportunities for structured, diabetes‐specific continuing education are more limited than in acute or hospital‐based settings.

Improving diabetes care in nursing homes also requires strengthening the educational pipeline. One promising strategy is to enhance the presence and quality of clinical training experiences in long‐term care settings. As Mueller et al. [39] emphasize, it is important for nursing programs to provide robust and meaningful clinical placements in nursing homes to help students develop competence, confidence, and interest in this field. Exposure to the complexity and rewards of caring for older adults with chronic conditions—such as diabetes—can help reposition nursing homes as viable and fulfilling career destinations. To achieve this, more nursing schools should actively build partnerships with long‐term care facilities and involve experienced nursing home staff in curriculum development and clinical teaching roles. Doing so may help address workforce shortages, reduce staff turnover, and elevate the quality of care through better‐prepared and more motivated graduates.

Although this study focused on diabetes management knowledge, the findings also highlight structural issues that influence care quality. In our sample, younger and less experienced professionals tended to report lower levels of diabetes‐specific training and are likely to face more precarious working conditions. Prior research has shown that more years of nursing experience are associated with greater job satisfaction and adaptation to the work environment, while instability and high workloads contribute to less person‐centered care [40].

The limited diabetes knowledge observed in this study should be interpreted within the broader organizational context of nursing homes. Precarious employment conditions, characterized by high workloads, staff turnover, and restricted opportunities for professional development, have been shown to negatively affect care quality and professional performance in long‐term care settings [40]. Such conditions may also constrain nurses’ ability to update and apply diabetes‐related knowledge in daily practice. Moreover, care in nursing homes involves multiple interrelated dimensions, including technical competence, communication, and individualized support, all of which are influenced by workforce stability and organizational context [41]. From this perspective, the knowledge deficits identified in our study may reflect not only individual educational gaps but also broader systemic challenges inherent to long‐term care environments. Addressing diabetes care in nursing homes therefore requires not only targeted education but also structural improvements that support stable staffing and ongoing professional development.

### 4.1. Limitations of the Study

While this study provides valuable insights into the diabetes management knowledge among nurses in nursing homes in Cadiz and Warsaw, several limitations must be acknowledged. First, the sample size, particularly in Warsaw, was relatively small, with only 57 nurses participating, which may limit the generalizability of the findings to other regions or countries. Second, the cross‐sectional design of the study captures knowledge at a single point in time, preventing any conclusions about changes in knowledge over time or the effectiveness of specific interventions. Third, the study did not evaluate additional factors that could influence diabetes knowledge, such as recent continuing education, access to educational resources, and institutional support, which may vary significantly between regions. Finally, efforts were made to minimize potential sources of bias in this study. All participants met predefined eligibility criteria and were recruited through a systematic approach within nursing homes in Cádiz and Warsaw, reducing the risk of selection bias. The adapted DMKAT‐14 questionnaire was culturally validated for both Spanish and Polish contexts, ensuring conceptual equivalence across settings. Data collection procedures were standardized, and participation was voluntary and anonymous, which likely reduced social desirability bias. Although no study is entirely free from bias, the design and methodology employed aimed to ensure reliability and comparability of results across both samples.

Future research should examine the extent and content of undergraduate clinical placements in nursing homes across countries, as differential exposure to long‐term care settings may contribute to observed gaps between formal education and diabetes‐related competencies.

## 5. Conclusion

This study found that diabetes management knowledge among nursing home nurses in both Cádiz and Warsaw shows room for improvement, particularly in areas critical to the care of frail older adults. Despite formal education and training, current knowledge levels may not fully meet the demands of diabetes care in long‐term care settings.

Improving diabetes care in nursing homes requires more than just updated education; it demands structural changes to support staff, reduce turnover, and ensure safe, person‐centered care. Strengthening nursing home‐specific training and improving work conditions are essential steps toward better outcomes and quality of life for institutionalized older adults with diabetes.

## 6. Implications for Nursing Practice, Research, Policy, and Education

The findings of this study have important implications across several domains. In nursing practice, the suboptimal knowledge scores among nursing home nurses in both Cádiz and Warsaw underscore the need for enhanced diabetes management competencies, particularly in recognizing and preventing hypoglycemia and adapting care to the frail, older population. Addressing these knowledge gaps is essential for promoting safer, more person‐centered diabetes care in long‐term care settings.

From a research perspective, this study highlights the value of cross‐national comparisons in identifying systemic and educational differences that may influence nursing competencies. Future research should explore the impact of tailored educational interventions, the integration of diabetes technologies such as CGM, and the influence of institutional support and work conditions on care quality.

In terms of policy, the lack of significant associations between formal training and diabetes knowledge calls for a reevaluation of current continuing education frameworks. Policymakers should consider mandating structured, evidence‐based diabetes training programs specific to long‐term care environments and incorporating clinical competencies related to geriatric diabetes management into national nursing standards.

Finally, the results carry implications for nursing education. Despite comprehensive undergraduate curricula in both countries, there appears to be a disconnect between academic preparation and the practical demands of diabetes care in nursing homes. Strengthening clinical placements in long‐term care facilities, involving experienced nursing home staff in teaching roles, and emphasizing applied knowledge in chronic disease management may help bridge this gap. These changes are critical to preparing nurses who are not only knowledgeable but also confident and motivated to care for one of the most vulnerable populations.

## Funding

This work was supported by a research internship grant from the ERASMUS program, the University of Cádiz, and the Medical University of Warsaw, which enabled the author Ana Domínguez‐Navarro to conduct research at the Medical University of Warsaw.

## Ethics Statement

The study received ethical approval from the Ethics Committee for Research in Cádiz, Spain (PEIBA0652‐N‐23), and the Ethics Committee of the Medical University of Warsaw (AKBE/66/2023). Informed consent was obtained from all participants. All procedures were conducted in accordance with the ethical standards of the Declaration of Helsinki.

## Consent

Informed and written consent was obtained from all individual participants included in the study. Participants were informed about the purpose of the study, and their participation was voluntary with the option to withdraw at any time without any consequences.

## Conflicts of Interest

The authors declare no conflicts of interest.

## Data Availability

The data that support the findings of this study are available from the corresponding author upon reasonable request. Due to privacy and ethical restrictions, the dataset is not publicly available. However, anonymized data may be shared with qualified researchers for scientific purposes.

## Appendix A

Strobe Checklist: Cross‐sectional study is presented in Table A1.

**TABLE A1:** STROBE checklist: cross‐sectional study.

Item no.	Recommendation	How addressed in the article
*Title and Abstract*		
1	Indicate the study’s design in the title or abstract	Title includes “Cross‐Sectional Study”; abstract summarizes design, objectives, methods, results, and conclusions

*Introduction*		
	Explain the scientific background and rationale	Detailed background on the challenges of diabetes care in nursing homes and the role of nurses.
	State specific objectives	Clearly stated: to compare diabetes management knowledge between Cádiz and Warsaw

*Methods*		
4	Present key elements of study design	Clearly described as a multicenter, observational cross‐sectional study
5	Describe setting, locations, and relevant dates	Conducted in nursing homes in Cádiz (Sept–Nov 2023) and Warsaw (Mar–Jun 2023)
6	Give eligibility criteria and methods of participant selection	Inclusion criteria: nurses with ≥ 6 months’ experience in diabetes care; purposive sampling
7	Clearly define all outcomes, exposures, and predictors	Primary outcome: DMKAT‐14 score; predictors: age, education, training, experience, exposure to diabetes
8	Provide data sources and measurement methods	Adapted DMKAT‐14 used; culturally validated in both countries; structured questionnaires for additional variables
9	Describe efforts to address potential sources of bias	Efforts to minimize bias included standardized data collection, cultural adaptation, and voluntary anonymous participation. Bias paragraph included in the Limitations section
10	Explain how study size was determined	Included nearly all eligible nurses; small Warsaw sample acknowledged as a limitation
11	Explain handling of quantitative variables	Described clearly; treated as continuous or categorical as appropriate
12	Describe all statistical methods	Descriptive statistics, *t*‐tests, ANOVA, and multiple linear regression reported. SPSS v24 used. Separate models for each city

*Results*		
13	Report numbers of participants at each stage	Final sample: 198 nurses in Cádiz, 57 in Warsaw
14	Give characteristics of participants	Full demographic and professional characteristics in Table 1
15	Report numbers of outcome events or summary measures	DMKAT‐14 mean scores reported by city, subgroup comparisons included
16	Give unadjusted and adjusted estimates with precision	Unadjusted comparisons (*t*‐tests, ANOVA) and adjusted regression models with *p* values and effect sizes reported
17	Report other analyses done	Regression analyses per city to identify predictors of knowledge. Effect sizes and non‐significant trends discussed

*Discussion*		
18	Summarize key results with reference to study objectives	Results interpreted in relation to knowledge gaps, training needs, and system‐level factors
19	Discuss limitations of the study	Limitations include sample size (Warsaw), cross‐sectional design, and contextual factors
20	Provide interpretation of results	Cautious interpretation; emphasizes implications for practice, education, and policy
21	Discuss generalizability	Findings are cautiously generalized; limitations acknowledged

*Other Information*		
22	Give the source of funding and role of funders	Acknowledgments state “This work was supported by a research internship grant from the ERASMUS program, the University of Cádiz, and the Medical University of Warsaw”

## References

[1] American Diabetes Association Professional Practice Committee , 13. Older Adults: Standards of Care in Diabetes-2025, Diabetes Care. (2025) 48, no. 1 Suppl 1, S266–S282, 10.2337/dc25-S013. 39651977 PMC11635042

[2] Munshi M. N. , Florez H. , Huang E. S. et al., Management of Diabetes in Long-Term Care and Skilled Nursing Facilities: A Position Statement of the American Diabetes Association, Diabetes Care. (2016) 39, no. 2, 308–318, 10.2337/dc15-2512, 2-s2.0-84962124070. 26798150 PMC5317234

[3] Idrees T. , Castro-Revoredo I. A. , Migdal A. L. , Moreno E. M. , and Umpierrez G. E. , Update on the Management of Diabetes in Long-Term Care Facilities, BMJ Open Diabetes Research and Care. (2022) 10, no. 4, 10.1136/bmjdrc-2021-002705. PMC9305812 35858714

[4] Moffet H. H. , Huang E. S. , Liu J. Y. et al., Severe Hypoglycemia and Falls in Older Adults With Diabetes: The Diabetes and Aging Study, Diabetes Epidemiology and Management. (2023) 12, 10.1016/j.deman.2023.100162. PMC10621321 37920602

[5] Szczerbińska K. , Topinková E. , Brzyski P. et al., The Characteristics of Diabetic Residents in European Nursing Homes: Results From the SHELTER Study, Journal of the American Medical Directors Association. (2015) 16, no. 4, 334–340, 10.1016/j.jamda.2014.11.009, 2-s2.0-84934441363. 25533147

[6] Durán Alonso J. C. , Prevalencia de Diabetes Mellitus en Pacientes Geriátricos Institucionalizados en la Provincia de Cádiz: Estudio Diagerca, Revista Española de Geriatría y Gerontología: Órgano Oficial de la Sociedad Española de Geriatría y Gerontología. (2012) 47, no. 3, 114–118. 10.1016/j.regg.2011.11.003 22578323

[7] Cebrián Cuenca A. M. and Escalada J. , Prevalencia de Obesidad y Diabetes en España: Evolución en Los Últimos 10 Años, Atención Primaria. (2024) 57, no. 3, 10.1016/j.aprim.2024.102992. PMC11459898 39326172

[8] Kalra S. and Sharma S. , Diabetes in the Elderly, Diabetes Therapy. (2018) 9, no. 2, 493–500, 10.1007/s13300-018-0380-x, 2-s2.0-85044527346. 29460258 PMC6104259

[9] Munshi M. N. , Sy S. , Lekarcyk J. , and Sullivan E. , A Successful Diabetes Management Model of Care in Long-Term Care Facilities, Journal of the American Medical Directors Association. (2021) 22, no. 6, 1322–1326.e2, 10.1016/j.jamda.2020.06.046. 32753320

[10] Vincent C. , Hall P. , Ebsary S. M. , Hannay BscPhm S. , Hayes-Cardinal BscPhm L. , and Husein N. , Knowledge Confidence and Desire for Further Diabetes-Management Education Among Nurses and Personal Support Workers in Long-Term Care, Canadian Journal of Diabetes. (2015) 40, no. 3, 226–233, 10.1016/j.jcjd.2015.11.001, 2-s2.0-84960971157. 26992287

[11] Gill E. A. , Corwin P. A. , Mangin D. A. , and Sutherland M. G. , Diabetes Care in Rest Homes in Christchurch, New Zealand, Diabetic Medicine. (2006) 23, no. 11, 1252–1256, 10.1111/j.1464-5491.2006.01976.x, 2-s2.0-33750176768. 17054604

[12] Farrer O. , Yaxley A. , Walton K. , and Miller M. , A Scoping Review of Best Practice Guidelines for the Dietary Management of Diabetes in Older Adults in Residential Aged Care, Primary Care Diabetes. (2019) 13, no. 4, 293–300, 10.1016/j.pcd.2019.02.005, 2-s2.0-85062639681. 30871835

[13] Stöhr D. , Mayer H. , and Soom Ammann E. , ‘After Mealtime Is Before Mealtime’—An Ethnographic Exploration of Nursing Home Residents’ Practices of Dealing With Meal Requirements, BMC Geriatrics. (2022) 22, no. 1, https://doi-org.bibliouca.idm.oclc.org/10.1186/s12877-022-03595-2. 10.1186/s12877-022-03595-2 PMC9783982 36550407

[14] Alotaibi A. , Al-Ganmi A. , Gholizadeh L. , and Perry L. , Diabetes Knowledge of Nurses in Different Countries: An Integrative Review, Nurse Education Today. (2016) 39, 32–49, 10.1016/j.nedt.2016.01.017, 2-s2.0-84961123581. 27006032

[15] Haugstvedt A. , Aarflot M. , Igland J. , Landbakk T. , and Graue M. , Diabetes Knowledge in Nursing Homes and Home-Based Care Services: A Validation Study of the Michigan Diabetes Knowledge Test Adapted for Use Among Nursing Personnel, BMC Nursing. (2016) 15, no. 1, 10.1186/s12912-016-0159-1, 2-s2.0-84978306113. PMC4928289 27366112

[16] von Elm E. , Altman D. G. , Egger M. , Pocock S. J. , Gøtzsche P. C. , and Vandenbroucke J. P. , STROBE Initiative. The Strengthening the Reporting of Observational Studies in Epidemiology (STROBE) Statement: Guidelines for Reporting Observational Studies, Lancet. (2007) 370, no. 9596, 1453–1457, 10.1016/S0140-6736(07)61602-X, 2-s2.0-36849065071. 18064739

[17] Modic M. B. , Vanderbilt A. , Siedlecki S. L. , Sauvey R. , Kaser N. , and Yager C. , Diabetes Management Unawareness: What Do Bedside Nurses Know?, Applied Nursing Research. (2014) 27, no. 3, 157–161, 10.1016/j.apnr.2013.12.003, 2-s2.0-84904120068. 24674695

[18] Wild D. , Grove A. , Martin M. et al., ISPOR Task Force for Translation and Cultural Adaptation. Principles of Good Practice for the Translation and Cultural Adaptation Process for Patient-Reported Outcomes (PRO) Measures: Report of the ISPOR Task Force for Translation and Cultural Adaptation, Value Health. (2005) 8, no. 2, 94–104, 10.1111/j.1524-4733.2005.04054.x, 2-s2.0-17144423549. 15804318

[19] Tristán-López A. , Modificación al Modelo de Lawshe Para el Dictamen Cuantitativo de la Validez de Contenido de un Instrumento Objetivo, Avances En Medición. (2008) 6, no. 1, 37–48, https://dialnet.unirioja.es/servlet/articulo?codigo=2981185.

[20] Lawshe C. H. , A Quantitative Approach to Content Validity, Personnel Psychology. (1975) 28, no. 4, 563–575, 10.1111/j.1744-6570.1975.tb01393.x, 2-s2.0-84986938722.

[21] Olsen M. T. , Rasmussen L. M. , Bach E. et al., Healthcare Professionals’ Competencies and Confidence in Managing Hospitalized Patients With Type 2 Diabetes, Diabetic Medicine: A Journal of the British Diabetic Association. (2024) 41, no. 9, 10.1111/dme.15392. 38924549

[22] Jensen M. K. , Houe S. M. M. , Bjerre-Christensen U. , Kingod N. R. , and Stenov V. , Needs Assessment for Developing a Train-the-Trainer Educational Program to Improve Healthcare Professionals’ Knowledge and Skills in Older Diabetes Care, Patient Education and Counseling. (2025) 138, 10.1016/j.pec.2025.109205. 40532570

[23] Albagawi B. , Alkubati S. A. , and Abdul-Ghani R. , Levels and Predictors of Nurses’ Knowledge About Diabetes Care and Management: Disparity Between Perceived and Actual Knowledge, BMC Nursing. (2023) 22, no. 1, 10.1186/s12912-023-01504-5. PMC10537144 37770877

[24] Song J. , Li S. , Gu C. et al., The Current Status and Influencing Factors of Diabetes Knowledge Among Non-Endocrinology Nurses of Tertiary General Hospitals: A Cross-Sectional Survey Study, BMC Nursing. (2025) 24, no. 1, 10.1186/s12912-025-02741-6. PMC11760706 39856658

[25] Lega I. C. , Kapur A. , Leung F. , and Zahedi A. , Type 2 Diabetes in Older Adults in Long-Term Care Homes: An Educational Intervention to Improve Diabetes Care, Canadian Journal of Diabetes. (2020) 44, no. 5, 407–413.e3, 10.1016/j.jcjd.2020.01.009. 32305292

[26] Pearce J. , Mann M. K. , Jones C. , van Buschbach S. , Olff M. , and Bisson J. I. , The Most Effective Way of Delivering a Train-the-Trainers Program: A Systematic Review, Journal of Continuing Education in the Health Professions. (2012) 32, no. 3, 215–226, 10.1002/chp.2114. 23173243

[27] Sinclair A. J. , Gadsby R. , Abdelhafiz A. H. , and Kennedy M. , Failing to Meet the Needs of Generations of Care Home Residents With Diabetes: A Review of the Literature and a Call for Action, Diabetic Medicine: A Journal of the British Diabetic Association. (2018) 35, no. 9, Advance online publication, 1144–1156, 10.1111/dme.13702, 2-s2.0-85051111929. 29873423

[28] Diabetes Canada Clinical Practice Guidelines Expert Committee , Meneilly G. S. , Knip A. et al., Diabetes in Older People, Canadian Journal of Diabetes. (2018) 42, no. Suppl 1, S283–S295, 10.1016/j.jcjd.2017.10.02. 29650107

[29] Laiteerapong N. , Karter A. J. , Liu J. Y. et al., Correlates of Quality of Life in Older Adults With Diabetes: The Diabetes and Aging Study, Diabetes Care. (2011) 34, no. 8, 1749–1753, 10.2337/dc10-2424, 2-s2.0-84862833218. 21636795 PMC3142040

[30] Raggi A. , Corso B. , Minicuci N. et al., Determinants of Quality of Life in Ageing Populations: Results From a Cross-Sectional Study in Finland, Poland and Spain, PLoS One. (2016) 11, no. 7, 10.1371/journal.pone.0159293, 2-s2.0-84979519307. PMC4951007 27434374

[31] Pigłowska M. , Kostka T. , and Guligowska A. , Do Determinants of Quality of Life Differ in Older People Living in the Community and Nursing Homes?, International Journal of Environmental Research and Public Health. (2023) 20, no. 2, 10.3390/ijerph20020916. PMC9858919 36673674

[32] Bouillet B. , Tscherter P. , Vaillard L. et al., Frequent and Severe Hypoglycaemia Detected With Continuous Glucose Monitoring in Older Institutionalised Patients With Diabetes, Age and Ageing. (2021) 50, no. 6, 2088–2093, 10.1093/ageing/afab128. 34324624

[33] Idrees T. , Castro-Revoredo I. A. , Oh H. D. et al., Continuous Glucose Monitoring-Guided Insulin Administration in Long-Term Care Facilities: A Randomized Clinical Trial, Journal of the American Medical Directors Association. (2024) 25, no. 5, 884–888, 10.1016/j.jamda.2024.01.031. 38460943 PMC11283256

[34] Gomez-Peralta F. , Abreu C. , Santos E. et al., A Telehealth Program Using Continuous Glucose Monitoring and a Connected Insulin Pen Cap in Nursing Homes for Older Adults With Insulin-Treated Diabetes: The Trescasas Study, Diabetes Technology and Therapeutics. (2025) 27, no. 5, 357–365, 10.1089/dia.2024.0356. 39587875

[35] Cappon G. , Vettoretti M. , Sparacino G. , and Facchinetti A. , Continuous Glucose Monitoring Sensors for Diabetes Management: A Review of Technologies and Applications, Diabetes and Metabolism Journal. (2019) 43, no. 4, 383–397, 10.4093/dmj.2019.0121, 2-s2.0-85072111660. 31441246 PMC6712232

[36] Ministry of Science and Higher Education , Komunikat Ministra Nauki I Szkolnictwa Wyższego Z Dnia 2 Czerwca 2015 R. W Sprawie Kryteriów I Trybu Oceny Czasopism Naukowych, 2015, no. 164, http://www.nauka.gov.pl/g2/oryginal/2015_06/57d62136155875b12419981aa086b9f9.pdf.

[37] Boletin Oficial del Estado , Orden CIN/2134/2008. Orden CIN/2134/2008, de 3 de Julio Por La Que Se Establecen Los Requisitos Para La Verificación de Los Títulos Universitarios Oficiales Que Habiliten Para El Ejercicio de La Profesión de Enfermero, 2008, 174, 31680–31683, https://www.boe.es/boe/dias/2008/07/19/pdfs/A31680-31683.pdf.

[38] Hu L. and Jiang W. , Assessing Perceptions of Nursing Knowledge, Attitudes, and Practices in Diabetes Management Within Chinese Healthcare Settings, Frontiers in Public Health. (2024) 12, 10.3389/fpubh.2024.1426339. PMC11345264 39188797

[39] Mueller C. A. , Alexander G. L. , Ersek M. , Ferrell B. R. , Rantz M. J. , and Travers J. L. , Calling all Nurses-Now is the Time to Take Action on Improving the Quality of Care in Nursing Homes, Nursing Outlook. (2023) 71, no. 1, 10.1016/j.outlook.2022.11.001. 36621418

[40] Fité-Serra A. M. , Gea-Sánchez M. , Alconada-Romero Á. et al., Occupational Precariousness of Nursing Staff in Catalonia’s Public and Private Nursing Homes, International Journal of Environmental Research and Public Health. (2019) 16, no. 24, 10.3390/ijerph16244921. PMC6950342 31817401

[41] Cohen-Mansfield J. and Parpura-Gill A. , Practice Style in the Nursing Home: Dimensions for Assessment and Quality Improvement, International Journal of Geriatric Psychiatry. (2008) 23, no. 4, 376–386, 10.1002/gps.1888, 2-s2.0-42449095589. 17726718

